# Cholera outbreaks: Public health implications, economic burden, and preventive strategies

**DOI:** 10.3934/publichealth.2025039

**Published:** 2025-08-05

**Authors:** Mona Gamal Mohamed, Eman Abdelaziz Ahmed Dabou, Shaimaa Abdelsamad, Shaimaa Hashem Elsalous

**Affiliations:** RAK College of Nursing, RAK Medical and Health Sciences University, UAE

**Keywords:** cholera outbreaks, public health, health implications, economic burden, preventive strategies, cholera prevention, global health challenges, outbreak management, health economics

## Abstract

**Background:**

Cholera remains a persistent and deadly global public health threat, with recent years witnessing a resurgence of large-scale outbreaks, particularly in conflict-affected and resource-limited regions. The disease disproportionately affects vulnerable populations lacking access to clean water, sanitation, and essential healthcare services.

**Objective:**

This systematic review aimed to synthesize evidence published between 2019 and 2024 to examine the evolving public health implications, economic burden, and prevention strategies associated with cholera outbreaks globally.

**Methods:**

Following the PRISMA (Preferred Reporting Items for Systematic Reviews and Meta-Analyses) guidelines, a comprehensive literature search was conducted across the major databases. Eligible studies were screened and assessed for quality and relevance using predefined inclusion criteria and standardized appraisal tools.

**Results:**

The review reveals an integrated perspective on cholera by analyzing the intersection of epidemiological trends, health system readiness, and socioeconomic vulnerabilities. Emerging factors such as climate change, population displacement, and political instability were identified as key contributors to cholera outbreaks. Innovative tools, including predictive modeling and artificial intelligence, demonstrate promise for early detection and response. The review also highlights the benefits and challenges of oral cholera vaccines (OCVs), the critical need for sustainable water and sanitation infrastructure, and the importance of community-based interventions.

**Conclusion:**

This review reinforces the urgency of adopting a multisectoral, systems-based approach to cholera prevention. Applying the “One Health” framework and aligning public health strategies with economic and policy insights can significantly enhance global efforts to reduce cholera's incidence and mortality. The findings inform key research and policy priorities to strengthen preparedness and resilience in high-burden settings.

## 1. Introduction

Cholera has been a significant global health threat for over two centuries, with seven major pandemics recorded since 1817. It remains a major public health concern, particularly in low- and middle-income countries and among vulnerable populations. Historically, cholera is estimated to have caused over 40 million deaths globally since the 19th century, and it continues to claim an estimated 21,000 to 143,000 lives each year worldwide, according to the World Health Organization (WHO) [1].

Cholera is an acute diarrheal illness caused by ingestion of food or water contaminated with the *Vibrio cholerae* bacterium. Transmission primarily occurs through the fecal–oral route, often in settings with inadequate sanitation, limited access to clean water, and overcrowded conditions. Once ingested, the bacteria colonize the small intestine and release the cholera toxin, leading to rapid dehydration and, if untreated, death [2].

The first cholera pandemic began in 1817 in India, spreading rapidly to Southeast Asia, the Middle East, Europe, and Eastern Africa. This initial outbreak was followed by six more pandemics, each with devastating consequences. The second pandemic (1826–1837) reached North America and Europe. The third (1846–1860) marked cholera's first appearance in South America. The fourth (1863–1875) spread to Naples, Spain, and the United States. The fifth pandemic (1881–1896) claimed over 250,000 lives in Europe and 50,000 in the Americas. The sixth pandemic (1899–1923) was especially deadly in India, Arabia, and North Africa [3].

The current seventh pandemic, which began in 1961 in Indonesia, differs from earlier pandemics that originated in the Ganges Delta. It spread across Asia in the 1960s, reached Africa in the 1970s—where cholera had not been reported for over 70 years and appeared in Peru in 1991, triggering widespread outbreaks across South and Central America [4].

In recent years, cholera has re-emerged with increasing frequency and severity. According to the WHO, between 2021 and 2023 alone, over 80 countries reported cholera cases, with more than 1.3 million cases and nearly 4000 deaths reported in 2022. A surge in cholera activity has been reported across multiple countries within the same timeframes, indicating widespread systemic vulnerabilities in the public health infrastructure. This resurgence highlights the urgent need for robust surveillance systems, improved water and sanitation services, and rapid response mechanisms to contain and prevent future outbreaks [5].

Cholera is primarily transmitted through contaminated water, with drinking water containing toxigenic *Vibrio cholerae* O1 or O139 being the most common source of infection [6]. Contaminated food, particularly raw or undercooked seafood, also plays a significant role in transmission. Additionally, poor sanitation and hygiene practices, such as inadequate sanitation facilities and insufficient hygiene, contribute to the spread of cholera [7].

Despite the global relevance of cholera, the existing literature has predominantly focused on either the epidemiological trends [8] or the biomedical aspects of the disease [9], with limited integration of its public health implications, economic burden, and preventive strategies into a single comprehensive framework. Several reviews have examined cholera outbreaks in isolated contexts or discussed prevention efforts regionally [10], while fewer studies have holistically analyzed the interplay of disease control, economic costs, and long-term policy measures. Therefore, this review aimed to (1) examine the public health implications of cholera outbreaks globally, (2) evaluate the economic impact of cholera on health systems and vulnerable populations, and (3) synthesize preventive strategies with an emphasis on global applicability and sustainability. The manuscript is structured to first provide a historical and epidemiological overview of cholera, followed by an analysis of its economic burden, and concludes with a critical appraisal of current and emerging prevention strategies.

## 2. Methods

This systematic review followed the PRISMA (Preferred Reporting Items for Systematic Reviews and Meta-Analyses) guidelines to ensure transparency, reproducibility, and methodological rigor. A systematic approach was employed to locate, select, extract, and synthesize relevant data to address the research question regarding cholera outbreaks, public health implications, the economic burden, and preventive strategies.

A comprehensive literature search was conducted to identify studies published between 2019 and 2024. The search was performed across the following electronic databases: PubMed, Scopus, Web of Science, and Google Scholar. These databases were chosen due to their broad coverage of health-related research and their relevance to the topics of cholera outbreaks and public health. The search terms included both medical subject headings (MeSH) and free-text keywords, such as “cholera outbreaks,” “public health,” “economic burden,” “preventive strategies,” and related synonyms. Boolean operators (AND, OR) were utilized to refine the search and combine these terms efficiently. For example, terms like “cholera” were combined with “economic burden” using AND, and synonyms for “preventive strategies” were linked with OR to ensure comprehensive coverage of the literature (Supplementary file).

**Figure 1.**
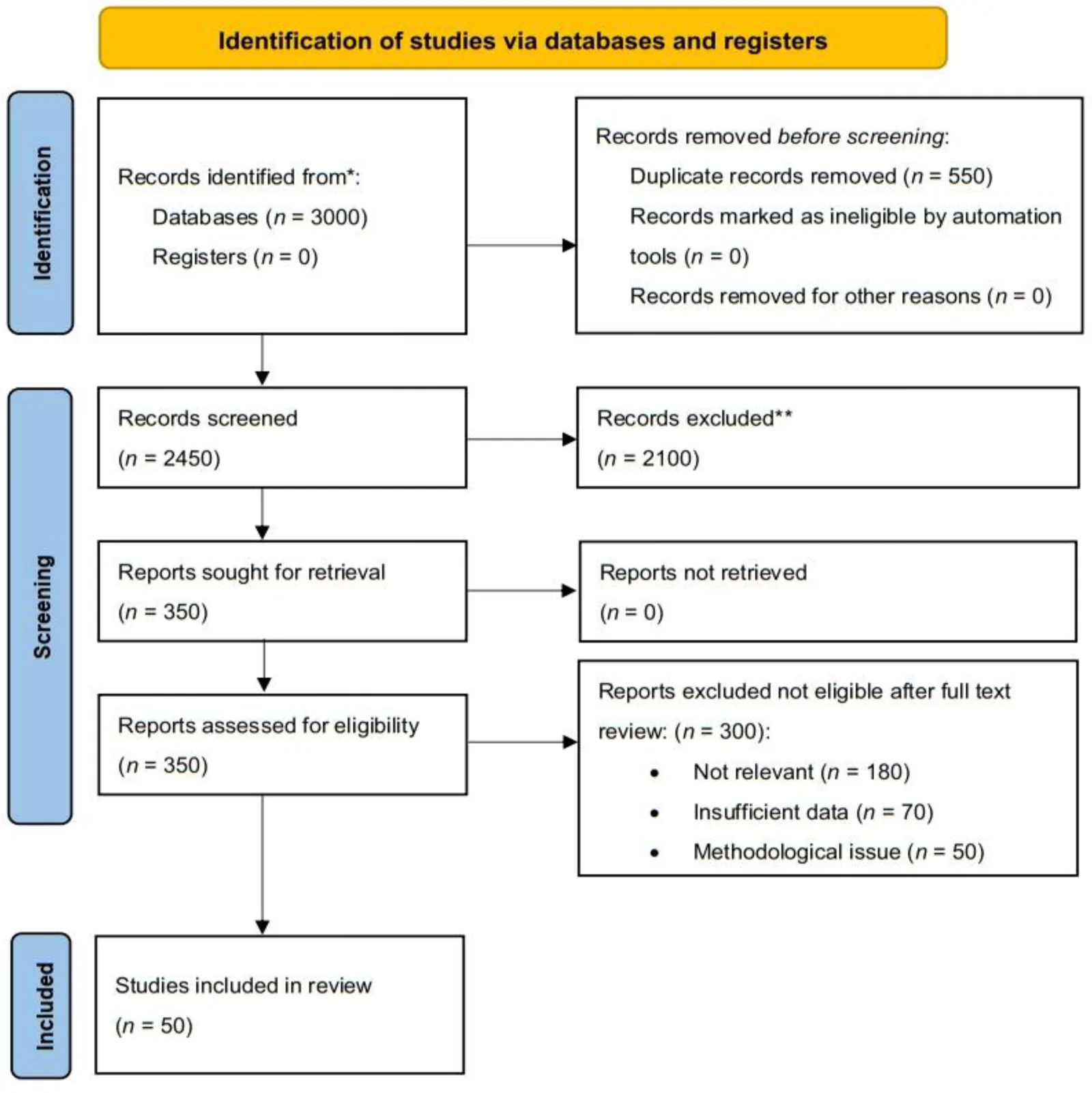
PRISMA 2020 flow diagram of study selection. From 3000 database records, 2450 were screened after removing duplicates. Following abstract and full-text screening, 50 studies met the inclusion criteria for the final review.

The search strategy was designed to maximize the retrieval of relevant studies while maintaining precision. The rationale for the chosen keywords was based on their relevance to the core concepts of cholera outbreaks, public health, and economic impact. We explicitly sought articles that provided insights into the epidemiology, economic costs, and preventive measures associated with cholera outbreaks. This search was designed to be exhaustive while adhering to the PRISMA guidelines, ensuring that all relevant studies were captured (Figure 1).

### 2.1. Inclusion and exclusion criteria

For the inclusion and exclusion criteria, see Table 1.

**Table 1.** Inclusion and exclusion criteria for study selection.

**Inclusion criteria**	**Exclusion criteria**
Studies published between 2019 and 2024.	Studies published before 2019.
Peer-reviewed articles published in English.	Articles not published in English.
Focus on cholera outbreaks, public health implications, economic burden, or preventive strategies.	Studies not focused on cholera or irrelevant to its public health, economic, or prevention aspects.
Quantitative, qualitative, or mixed-methods designs, including observational studies, randomized controlled trials (RCTs), and reviews.	Non-peer-reviewed publications, including editorials, opinion pieces, or conference abstracts.
Discussion of economic impact, healthcare costs, or cholera control strategies.	Articles related to other infectious diseases without direct relevance to cholera.
Studies involving human populations or healthcare systems affected by cholera.	Studies lacking sufficient data, a clear methodology, or defined research questions.

### 2.2. Screening and selection process

The screening process was carried out in two stages to ensure the relevance and quality of the studies included in the review. Initially, a comprehensive search was conducted to identify records from the specified databases. The total number of records identified was 3000, with duplicates removed during the screening process, resulting in 2450 records for further evaluation.

Two independent researchers performed the screening of titles and abstracts. Any discrepancies in the assessment of the articles were resolved through discussion, and a third reviewer was consulted if required to ensure consistency and accuracy. The records were screened for relevance according to the inclusion and exclusion criteria. In total, 500 articles were assessed for full-text eligibility after the initial screening.

In the second stage, the full-text articles were carefully reviewed for eligibility. In total, 100 articles were deemed suitable for inclusion on the basis of the criteria, and a final selection of 50 articles was included in the review after confirming their relevance and quality.

### 2.3. Data extraction and synthesis

Data were extracted from the selected articles using a standardized form to capture relevant information such as the study design, sample size, geographical location, methodology, and outcomes related to public health implications, economic burden, and preventive strategies. The extracted data were then synthesized, and key themes were identified across studies.

### 2.4. PRISMA adherence

This review strictly adhered to the PRISMA guidelines, which include transparency in the search process, selection criteria, and data synthesis methods. The PRISMA flowchart was used to visually represent the progression of records through the phases of the systematic review (identification, screening, eligibility, and inclusion).

## 3. Results

### 3.1. Recent cholera outbreaks and affected regions

Globally, cholera deaths have increased significantly, with annual fatalities rising from 83,045 in 1990 to 117,167 in 2019, underscoring the urgent need for improved public health measures [11]. The WHO reported that, as of 1 February 2023, at least 18 countries were actively reporting cholera cases. Recent major outbreaks have highlighted the ongoing challenges posed by cholera [12]. For instance, the African region has been particularly affected, accounting for 79.2% of global cholera deaths in 2019 [13]. Countries with high mortality rates included Nigeria, the Central African Republic, Eritrea, and Botswana. The Eastern Mediterranean region accounted for 13% of global cholera deaths, while South Asia, historically a hotspot for the disease, continues to face regular outbreaks [14].

The cholera outbreak in Haiti, which began in 2010, serves as a critical example of how multiple factors can converge to exacerbate the spread of infectious diseases. The outbreak was linked to UN peacekeepers from Nepal, who introduced the bacteria into the country [15]. However, the rapid spread of cholera was not solely due to the introduction of the pathogen but was facilitated by a complex array of factors. These included the devastation caused by the 2010 earthquake, which severely damaged the country's infrastructure, including water and sanitation systems. The lack of access to clean water, inadequate sanitation, and the absence of prior immunity within the population created a perfect storm for widespread transmission. These conditions highlight how environmental, social, and public health vulnerabilities can interact, leading to a large-scale and protracted cholera epidemic. The complexity of this outbreak underscores the importance of addressing structural and sociopolitical factors in understanding the persistence of cholera as a global health challenge [16]. Meanwhile, the ongoing civil war in Yemen triggered the world's largest modern cholera epidemic, with Yemen accounting for 84% of global cholera cases in 2017 and 93% in 2019 [17].

Since the last update, new cholera cases and deaths have been reported across various regions, with Africa remaining the most severely affected. In Burundi, from 28 July to 31 August 2024, 41 new cases and two deaths were reported, bringing the total to 687 cases and three deaths since 1 January 2024, compared with 609 cases and nine deaths in 2023. Comoros reported 13 new cases since 28 July 2024, with a total of 10,342 cases and 149 deaths since the start of the year; notably, no cases were reported in 2023 [18]. The Democratic Republic of the Congo saw a concerning rise, with 1527 new cases and 30 deaths from 28 July to 31 August 2024, bringing the total to 23,291 cases and 337 deaths for the year, compared with 24,121 cases and 222 deaths reported in 2023. In Ethiopia, 1743 new cases and 25 deaths were reported, totaling 23,030 cases and 207 deaths since 1 January 2024, up from 17,796 cases and 220 deaths in 2023 [19].

Other African countries are also facing significant challenges with cholera outbreaks. In Ghana, 24 new cases were reported in 2024, while Kenya reported five new cases, bringing the total for the year to 300 cases and three deaths. Mozambique documented 12 new cases, reaching a total of 8183 cases and 17 deaths in 2024 [18]. Nigeria experienced a significant increase in cases, with 3142 new cases and 94 deaths reported in 2024, bringing the total to 5951 cases and 176 deaths for the year. In Somalia, 1649 new cases and four deaths were reported in 2024, totaling 18,218 cases and 138 deaths. Sudan reported 395 new cases and 37 deaths in 2024, bringing the total to 2803 cases and 37 deaths for the year. Togo confirmed 10 new cases and one death in 2023, maintaining a total of 10 cases [20].

In the Americas, Haiti reported 181 new cases and one death since 27 July 2024, totaling 9659 cases and 142 deaths for the year, a decrease from 38,036 cases and 421 deaths in 2023 [15]. In Asia, Afghanistan has seen a dramatic rise, reporting 30,170 new cases and 12 deaths since 27 July 2024, with a total of 125,471 cases and 60 deaths for the year [21]. Bangladesh recorded 98 new cases, totaling 168 cases for 2024, while China reported four new cases. Nepal had 38 new cases, while Pakistan documented 10,983 new cases, bringing the total to 49,619 cases this year. Syria reported 143 new cases, with a total of 10,563 cases, while Yemen saw 10,656 new cases and 21 deaths, totaling 31,809 cases and 153 deaths in 2024 [22]

**Figure 2.**
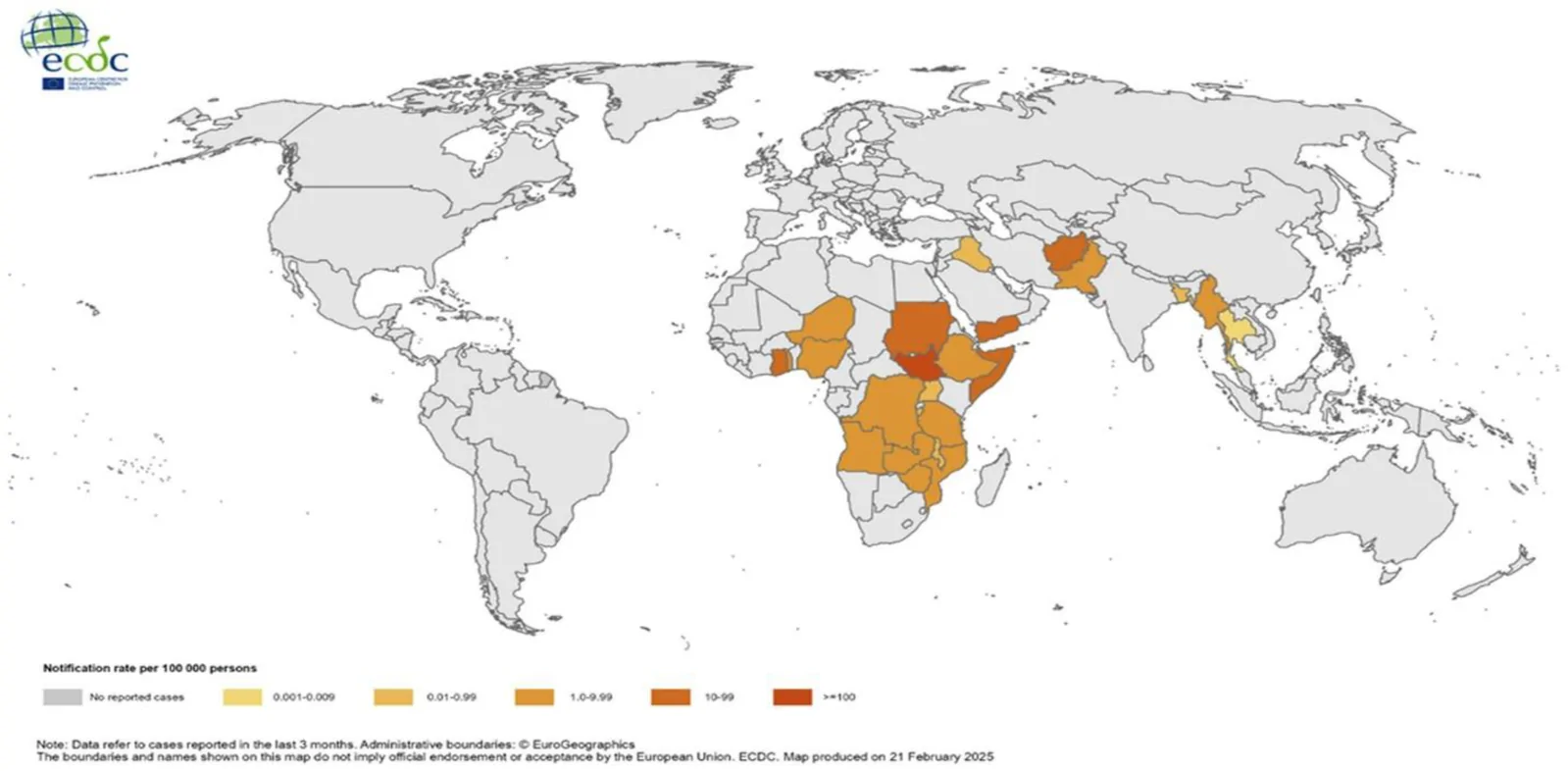
Geographical distribution of cholera cases reported worldwide from June 2024 to February 2025. Global cholera notification rates per 100,000 persons (as of February 2025). This map illustrates cholera notification rates based on cases reported in the last 3 months. Data are sourced from the European Centre for Disease Prevention and Control (ECDC). Administrative boundaries © Euro Geographics. Map produced by ECDC on 21 February 2025. Source: European Centre for Disease Prevention and Control. Communicable Disease Threats Report – Week 8, 2025. Available from: https://www.ecdc.europa.eu.

**Figure 3.**
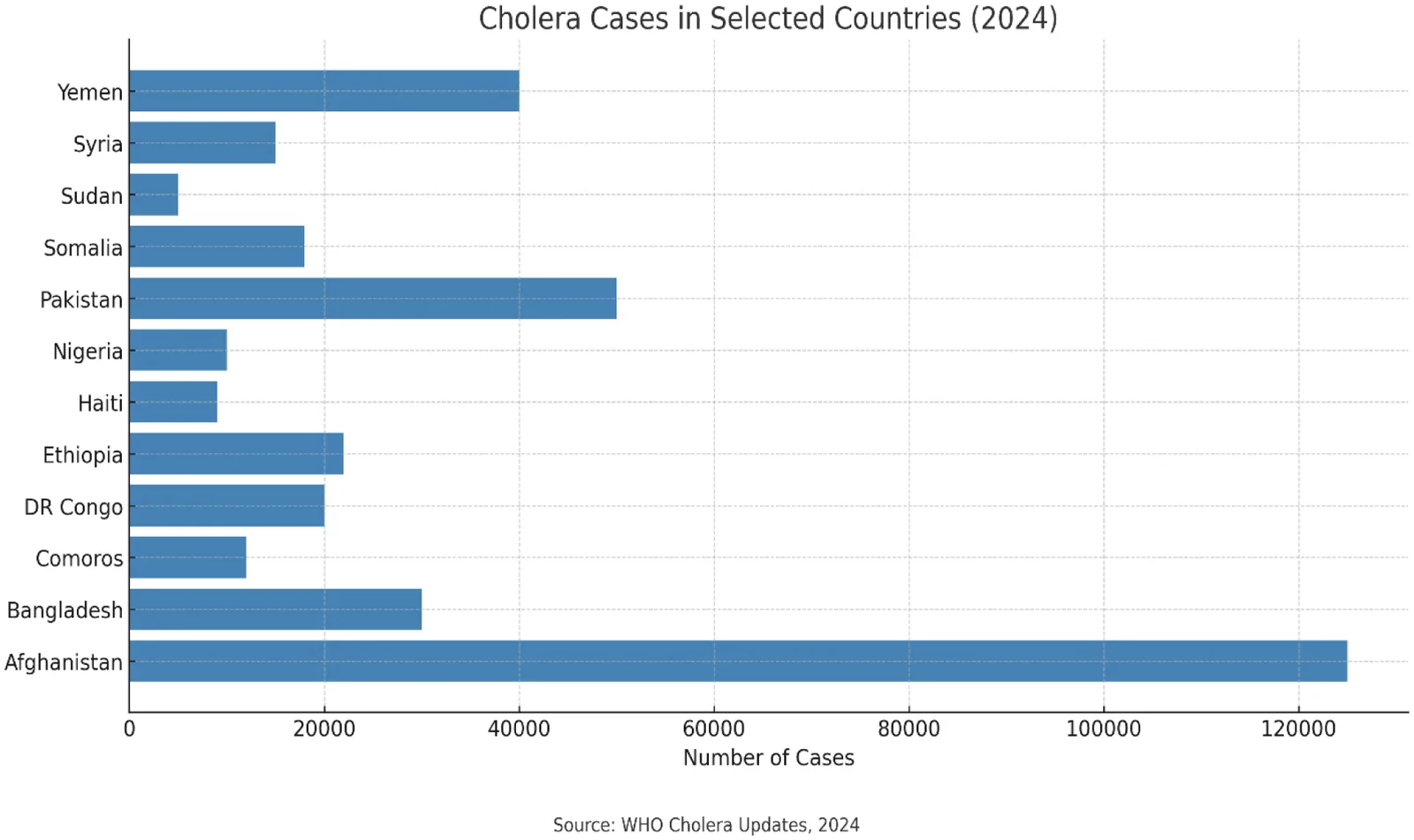
Cholera burden in 2024: Reported cases across conflict-affected and high-risk countries. Afghanistan reported the highest number of cholera cases, followed by Pakistan, Yemen, and Bangladesh. Data reflect WHO Cholera Updates, 2024.

In Europe, cholera cases remain minimal; in 2022, nine European Union (EU)/ European Economic Area (EEA) reported 29 cases, a slight increase from two cases in 2021 and none in 2020. The 2019 report noted 25 cases in EU/EEA countries, all with travel histories to cholera-affected areas [9],[23]. (Figure 2 and Figure 3).

Key risk factors include eating unwashed vegetables or fruits, storing water in containers, drinking from public wells, using public toilets, and purchasing water products in sachets from street vendors. Direct household exposure and traveling to affected areas further heighten the risk, particularly in regions lacking adequate tap water. Vulnerable populations include individuals with low gastric acidity, those with blood Type O (who are at greater risk for disease), humanitarian aid workers, refugees, internally displaced people, and travelers to endemic or outbreak areas [24].

There is growing evidence that climate change, particularly extreme weather events such as floods, droughts, and rising temperatures, is increasingly contributing to the persistence and resurgence of cholera and other waterborne diseases [25]. These climate-related extremities significantly disrupt sanitation systems, limit access to safe drinking water, and compromise public health infrastructure. In Africa alone, the population is expected to double by 2050 and quadruple by 2100 [26], placing immense pressure on already strained water and sanitation resources. Rapid urbanization, coupled with inadequate WASH (water, sanitation, and hygiene) services, further amplifies vulnerability to cholera transmission in densely populated, low-resource settings. Without timely, climate-resilient interventions, the risk of recurrent outbreaks will continue to escalate. As highlighted in recent literature [27], addressing the complex interplay between climate stressors and public health requires integrative strategies that combine environmental surveillance, adaptive infrastructure planning, and robust epidemiological data collection systems [28].

### 3.2. Public health implications

Cholera outbreaks result in high morbidity and mortality, especially among susceptible individualsm including children below five and immunocompromised individuals [29]. The rapid onset of cholera can overwhelm communities, particularly in low-resource settings. Cholera causes significant fluid and electrolyte loss, which can lead to dehydration. If left untreated, this can progress to hypovolemic shock and malnutrition, further worsening health outcomes. Prolonged dehydration episodes can also result in chronic kidney disease or other complications, such as irritable bowel syndrome (IBS) [30]. Additionally, malnourished children may experience stunted growth and developmental delays or become more susceptible to other infections, perpetuating a cycle of poor health. The case fatality rate exceeds 50% without proper clinical management [31].

Uncontrolled cholera outbreaks, endemicity, and repeated outbreaks are devastating to communities and their prospects for development. Cholera outbreaks place a substantial strain on healthcare systems, particularly in low-income countries. Cholera infections do not occur by chance; rather, cholera affects communities already burdened by conflict, a lack of infrastructure, poor health systems, malnutrition, or other political issues and wars [28].

Cholera outbreaks in conflict-affected countries within the Eastern Mediterranean Region (EMR) such as Sudan, Syria, Yemen, and Palestine underscore the profound impact of political instability, displacement, and the collapse of health infrastructure on disease transmission. In Sudan, ongoing armed conflict and mass displacement have severely disrupted access to clean water and sanitation, leading to widespread cholera outbreaks, with UNICEF reporting over 5000 suspected cases in 2023 alone [32]. In Yemen, the situation remains dire, with the WHO identifying it as having the highest burden of cholera globally due to prolonged war, collapsed healthcare services, and widespread famine [33]. Syria continues to face repeated cholera outbreaks amid infrastructural damage and disrupted water treatment systems, particularly in the northeast [34]. Similarly, in Palestine, the blockade and recurrent conflict have resulted in limited access to potable water and sanitation, creating a fertile ground for waterborne diseases such as cholera [35]. These cases exemplify the intersection of conflict and disease, highlighting how weakened governance, compromised health systems, and humanitarian crises exacerbate vulnerability to cholera transmission.

In regions already struggling with limited health resources, the sudden influx of cholera patients can overwhelm hospitals and clinics. This can lead to delays in treatment for cholera patients and other nonepidemic-related healthcare needs. Healthcare facilities in cholera-endemic areas often face challenges such as shortages of health supplies, inadequate staffing, and a lack of isolation facilities, all of which hinder effective response efforts. Moreover, cholera outbreaks disrupt the regular functioning of healthcare systems by diverting resources and personnel to contain the disease [28],[36].

Cholera's impact on population health, healthcare infrastructure, and long-term health outcomes for survivors is far-reaching. Addressing cholera requires not only immediate medical interventions but also long-term investments in clean water access, sanitation infrastructure, and strengthening of the healthcare system [37]. Efforts to control the global burden of cholera will continue to require coordinated actions across public health, governmental, and international sectors to prevent outbreaks and improve outcomes for survivors [38].

### 3.3. Economic burden

The overall global burden of cholera is poorly understood because only a small fraction of cases is reported to the WHO. Underreporting arises because of weak or absent surveillance systems, although countries also face disincentives for reporting cases, such as risks to tourism and export industries [39].

The economic burden caused by cholera outbreaks is complex and demands urgent global attention, particularly in fragile settings. In Syria, the outbreak has intensified existing challenges stemming from prolonged armed conflict, water insecurity, climate change, political instability, and the aftermath of a devastating earthquake. These interlinked crises have not only facilitated the rapid spread of cholera but have also severely undermined the country's response capacity. Disruptions to healthcare services, poor sanitation infrastructure, and water scarcity have amplified the transmission of the disease. Moreover, limited national resources are being diverted to manage the crisis, placing further strain on an already fragile economy. The outbreak has increased public health expenditures and disrupted livelihoods, underscoring the need for coordinated international support to mitigate both the health and economic consequences [10].

**Figure 4.**
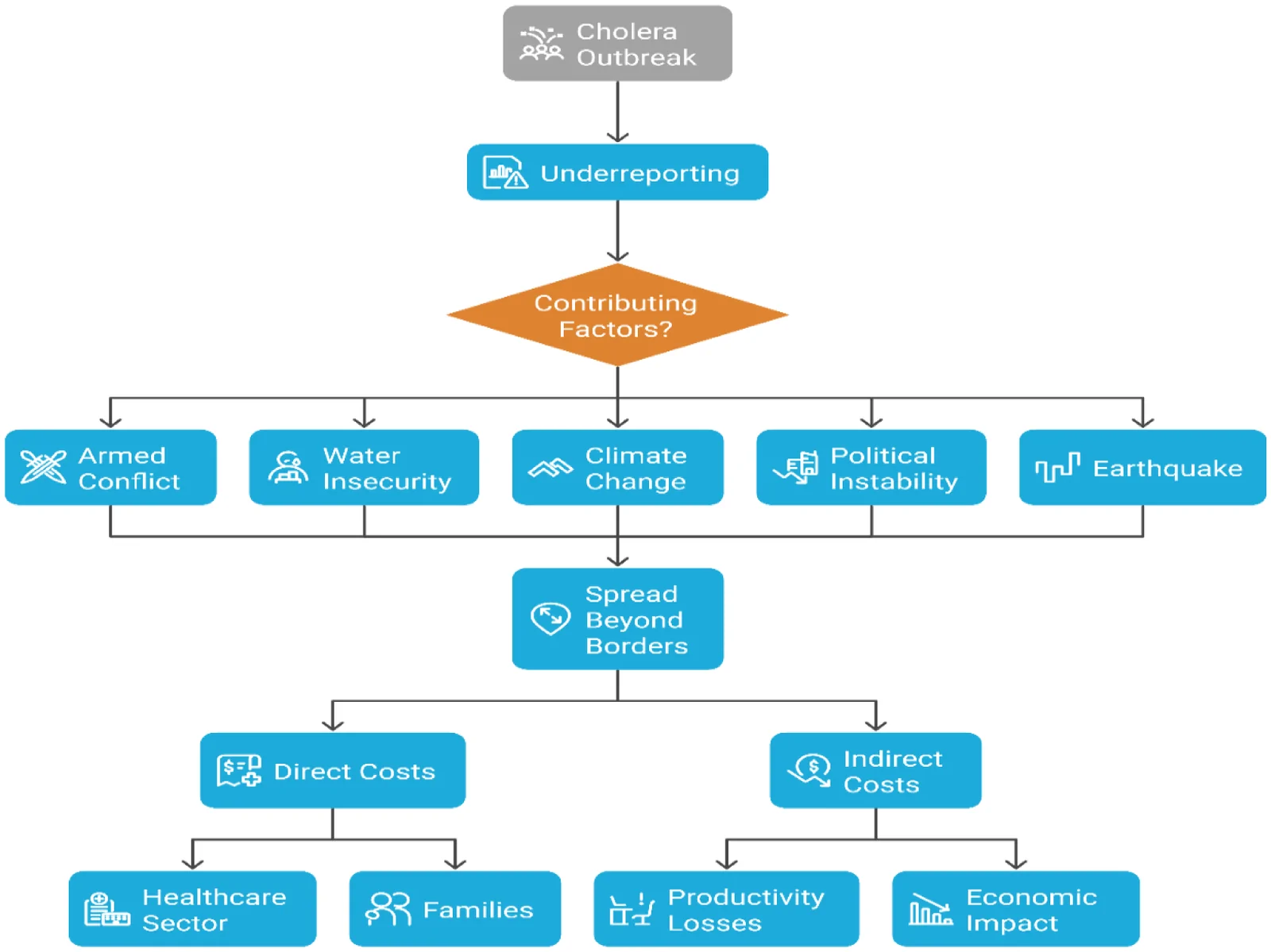
*Socioeconomic cascade of cholera outbreaks and contributing factors*. The diagram illustrates how underreporting, driven by conflict, climate change, water insecurity, and other crises, facilitates cross-border transmission and generates both direct (e.g., healthcare, household) and indirect (e.g., productivity, economic) costs. This underscores cholera as a multisectoral challenge requiring coordinated responses beyond the health sector.

The direct expenses of treating cholera cases are generally low, typically less than $100 per case in most settings. The costs include those for patients, their families, and lost productivity, as well as for the healthcare sector [40]. However, the estimated cost increases dramatically to more than $1000 per case when the larger socioeconomic impact is considered, including productivity losses caused by fatalities or the overall impact on the economy [41]. The flowchart below maps how outbreaks, driven by upstream vulnerabilities, result in both direct (e.g., healthcare system strain, burden on families) and indirect costs (e.g., reduced productivity, broader economic downturn). (Figure 4)

### 3.4. Preventive strategies

Advocacy strategies aimed at combating cholera must include evidence-based policy outreach and effective communication tailored to the specific challenges of the disease [42]. These initiatives are crucial for changing public and stakeholder perceptions, expediting decision-making, and securing funds to address shortages in cholera prevention and treatment programs. Given the complex factors that contribute to cholera outbreaks such as water insecurity, climate change, and political instability, advocacy efforts must also focus on raising awareness of these underlying causes. A deliberate, context-specific approach can increase public support and visibility, ensuring that cholera receives the attention it urgently needs to reduce its health and economic impact [43].

Cholera prevention requires a multisectoral approach, including community engagement, where individuals participate in developing and implementing programs [44]. Innovative technology, affordable sanitation solutions, and a cooperative approach combining governments, international organizations, the private sector, and local communities are essential [31]. Additionally, preventive measures such as improving water, sanitation, and hygiene (WASH), vaccination programs, and the use of oral cholera vaccines (OCV) are crucial to reducing the global burden of cholera. Developing outbreak response programs and increasing communication regarding cholera's prevention, risks, and symptoms are critical for mitigating future outbreaks [45].

The WHO brings together more than 50 organizations under the Global Task Force on Cholera Control (GTFCC) to provide a strong framework to reduce cholera deaths by 90% by 2030 and eliminate cholera in as many as 20 countries by 2030 [3],[39]. Sustained control will necessitate thorough and effective cholera surveillance, quick diagnosis, treatment, and health education. Ultimately, political will at all governmental levels is required to achieve this goal [31],[46].

Coordinated multisectoral action, community engagement, enhanced surveillance systems, long-term infrastructure rebuilding, and securing sustainable funding are crucial to reducing future vulnerability to cholera outbreaks [47]. To reduce vulnerability to future cholera outbreaks, coordinated multisectoral actions should prioritize community engagement, enhanced surveillance systems, long-term investments in water and sanitation infrastructure, and sustainable funding mechanisms [48],[49].

Following cholera outbreaks in Iraq, Lebanon, and Syria, Jordan implemented measures to prevent cross-border spread. The government-imposed travel and trade restrictions to control disease importation. The Ministry of Health led the national health response, with the Jordanian Center for Disease Control coordinating efforts under the National Cholera Preparedness and Response Plan [50],[51]. Given the vulnerability of refugee camps, platforms like the Health Sector Working Group facilitated information sharing and coordination. In December 2022, a whole-of-government risk assessment, including a field visit to the Zaatari refugee camp, identified key priorities, leading to the formation of a risk communication group and case management training [52].

OCV is regarded as a crucial part of an integrated control package, together with WASH, to control cholera in endemic or outbreak scenarios at the household and communal levels [53]. Although long-term investments to improve water and sanitation systems are ultimately necessary for cholera elimination, vaccinations serve as a cost-effective complementary strategy to reduce and control the burden of cholera and other infectious waterborne diseases in the short and medium term [45]. Hsiao et al. concluded that cholera vaccination is a practical short- to medium-term option recommended by the WHO, and it can still be a feasible, cost-effective plan, especially as the costs of building the WASH infrastructure are considerably higher for countries facing a significant cholera burden. However, WASH may be the more cost-effective solution in the long term when implemented properly [36],[54],[55].

While OCVs are a critical component of cholera prevention strategies, their effectiveness is often limited by barriers to acceptance at the community level. Cultural beliefs, religious misconceptions, vaccine-related misinformation, and lack of trust in public health systems can hinder vaccine uptake, particularly in underserved or conflict-affected areas [56]. In some regions, communities may perceive OCV campaigns as foreign-driven or unnecessary if the perceived risk of cholera is low. Low health literacy and past experiences with ineffective interventions can further reinforce hesitancy. To improve acceptance, it is essential to engage local leaders, community health workers, and trusted influencers to deliver culturally sensitive education and address fears proactively. Tailoring communication strategies to specific social and linguistic contexts can significantly enhance vaccine confidence and coverage [57].

The costs per cholera case were found to be relatively low: $1000/case. There is adequate evidence to support the economic value of vaccination for the prevention and control of cholera when targeted at high-incidence populations and/or areas with high case fatality rates due to cholera. When herd immunity is considered, vaccination also becomes a cost-effective option for the general population and is comparable in cost-effectiveness with other routine immunizations programs [58],[59].

One of the advantages of mass vaccination campaigns is providing indirect protection, as vaccinated individuals shed less *Vibrio cholerae* into water sources, reducing transmission to unvaccinated individuals. This community-level protection further enhances the vaccine's effectiveness and underscores the importance of widespread vaccine distribution in cholera-endemic areas. However, the growing gap between vaccine supply and demand highlights the need to expand production and distribution efforts to better support global cholera prevention and control strategies [60],[61].

Worldwide stakeholders have long advocated for targeted stockpiles of OCVs in high-risk areas to enable rapid outbreak containment. In response, a global OCV stockpile was established in 2011 to support mass vaccination campaigns during outbreaks. As of the end of 2024, the demand for OCVs has surged dramatically, with over 140 million doses requested between 2013 and 2024, yet only approximately 60% of these requests were fulfilled, reflecting recurring supply shortages across multiple deployments. This growing gap between demand and supply has led to major constraints: since 2022, preventive vaccination campaigns have largely been suspended, and in outbreak responses, only one of the recommended two doses is currently administered. These shortfalls highlight the urgency of scaling up global vaccine production. In this context, establishing regional or national stockpiles is increasingly discussed as a complementary strategy to the global mechanism. Such localized reserves could enhance timely responses and logistical efficiency; however, they also raise concerns about equitable access, sustainability, and coordination with the existing global stockpile infrastructure [62].

Artificial intelligence (AI) and predictive modeling hold significant promise in enhancing cholera prevention efforts, particularly through early warning systems and forecasting outbreaks. For instance, in Bangladesh, AI-driven tools have been piloted to integrate climate data, water quality indicators, and historical disease trends to predict cholera hotspots and guide targeted interventions. Similarly, the WHO-supported Diarrheal Disease Surveillance System in Africa is exploring machine learning models to strengthen real-time outbreak detection. However, the implementation of these innovations in resource-limited settings remains challenging due to constraints such as unreliable internet connectivity, limited digital infrastructure, insufficient technical expertise, and lack of integration with the existing health systems. Addressing these barriers requires sustained investment in digital health infrastructure, capacity-building for health workers, and policies that foster data sharing and interoperability [63].

### 3.5. Cholera management and treatment

Cholera necessitates prompt medical intervention; failure to do so can result in fatality. It is essential to accurately evaluate the presence of dehydration and malnutrition, and to identify any other coexisting health conditions in the management of cholera. Fluid replacement is an extremely effective treatment to decrease mortality [56]. Most patients with mild to moderate cholera can be managed with an oral rehydration solution (ORS). Rice-based ORSs decrease the amount of stool lost in severe cholera. Locally prepared solutions such as “Dhaka's solution,” which contains more potassium, and Ringer's Lactate, a suitable polyelectrolyte fluid, can also be used. Patients with cholera usually require 200 mL/kg of intravenous fluid replacement in the first 24 hours of therapy. An ORS should be used alongside intravenous therapy as it is richer in electrolytes and glucose than standard intravenous therapy [64],[65].

Numerous antibiotics, including doxycycline, tetracycline, ciprofloxacin, co-trimoxazole, erythromycin, and azithromycin, have demonstrated efficacy in the treatment of cholera. In many nations, doxycycline is recommended as the primary treatment for adults, while azithromycin is preferred for pregnant women and children [64].

The WHO Guidelines for Cholera Management outline the following treatment steps for patients diagnosed with cholera: First, assess the level of dehydration upon the patient's arrival at the hospital. Next, initiate rehydration in two distinct phases: the rehydration phase, lasting 2 to 4 hours, followed by the maintenance phase, which continues until the diarrhea subsides [66]. The intravenous route should be utilized exclusively in the following circumstances: (A) For severely dehydrated patients, in whom an infusion rate of 50–100 mL/kg/h is recommended during the rehydration phase; (B) for moderately dehydrated patients who are unable to tolerate oral rehydration; and (C) during the maintenance phase for patients classified as high stool purgers (greater than 10 mL/kg/h) [67]. It is essential to maintain hydration by replacing fluid losses until the diarrhea ceases. In the maintenance phase, an ORS should be administered at a rate of 800–1000 mL/h, unless the patient is a high stool purger, in which case, the intravenous route is advised. Additionally, an oral antibiotic should be provided to patients experiencing moderate or severe dehydration. Finally, patients may be discharged when they demonstrate oral tolerance of 1000 mL/h or more, a urine output of 40 mL/h or greater, and a stool output of 400 mL/h or less [8],[68],[69].

### 3.6. Challenges in cholera prevention and control

Restoration of weak infrastructures in many countries in the region, such as Afghanistan, Iraq, Somalia, and Yemen, requires long-term political commitment and significant investment. Additionally, massive population movements due to protracted political instability and acute conflicts has damaged the WASH and health infrastructure and compromised immunization efforts. These factors have weakened the preparedness and response capacities for controlling cholera epidemics [70]. Moreover, inadequate health literacy, the absence of national plans, inappropriate clean water supply, inadequate cross-border collaborations, insecurity, and large population movements within the region add pressure to the existing weak social services (i.e., water and sanitation), creating challenges in overcoming the spread of cholera [71],[72].

It is critical to note the challenges faced by healthcare workers, such as compounded health system issues; insufficient resources for prevention, cholera treatment, and case identification; and loss of motivation among nurses due to uncertainty about salaries and inadequate healthcare supplies in some countries [73]. Factors that could worsen the situation in the coming years include climate change, urbanization, the rise of social inequalities, and increased population density [74],[75].

Current challenges in managing cholera in Africa include limited clinical recognition of cholera at the early stages, often referred to as a low index of suspicion, particularly in areas where cases are sporadic or symptoms overlap with other diarrheal diseases. Additional barriers include inadequate community-based surveillance systems, poor health-seeking behaviors that prevent timely presentation to health facilities, insufficient numbers of cholera treatment centers (CTCs) and cholera treatment units (CTUs) to deliver standardized clinical care, delays in mobilizing national and international response teams, a shortage of trained healthcare workers, and bureaucratic delays in issuing visas for technical experts [76]. Additionally, significant shortages and delays of health supplies can lead to preventable and avoidable deaths. Antibiotic resistance, inadequate healthcare funding, and poor WASH infrastructure, as well as cultural beliefs that result in some groups refusing medical interventions, further compound these challenges [77],[78].

Reaching vulnerable populations such as refugees and internally displaced people (IDP) remains a major challenge in cholera prevention. These groups often live in overcrowded, poorly resourced settings with limited access to safe water, sanitation, and healthcare. Targeted interventions such as mobile health services, community health workers, and tailored WASH strategies are essential. Culturally appropriate education campaigns and collaboration among governments, nongovernmental organizations (NGOs), and humanitarian agencies are critical to ensure that displaced populations are included in national cholera preparedness and response plans [79].

### 3.7. Global and regional collaboration

The WHO maintains a public database of cholera cases and releases an annual summary of national data in the Weekly Epidemiological Record, along with updates on outbreaks. The Global Task Force on Cholera Control (GTFCC) of the WHO has led an initiative titled Ending Cholera: A Global Roadmap to 2030, which aims to reduce cholera-related mortality by 90% by the year 2030, using 2017 as the baseline. This global strategy targets 47 cholera-endemic countries and emphasizes coordinated efforts across the health and WASH sectors. By concentrating resources in the highest-burden areas, the roadmap seeks to save lives, promote equity, and reduce the significant economic impact of cholera and related enteric diseases [77].

The strategic priorities to overcome cholera include the swift identification of cholera cases and prompt reactions to epidemics using an early warning surveillance system (EWARS) paired with improved laboratory culture capabilities and specialized healthcare centers for cholera treatment. Another priority is preventing cholera's recurrence in identified hotspots by improving WASH and delivering OCVs. A third strategy is developing a well-organized network to provide financial support to countries where cholera is endemic. This network also brings national and international collaborators together to promote intersectoral coordination, supply mobilization, technical assistance, and cooperation to control cholera [80],[81].

In 2017, the GTFCC introduced a global roadmap with the goal of reducing cholera mortality by 90% and eliminating the disease in at least 20 countries by 2030. The GTFCC, through its technical working groups and in collaboration with cholera-affected countries, coordinates health, WASH, and logistical resources to implement this plan. The strategy emphasizes three key pillars: early detection and rapid response to outbreaks, targeted preventive measures including OCV in high-risk areas, and the effective mobilization and management of human, technical, and financial resources [82]. Additionally, the One WASH program was launched to support over 20 cholera-affected countries, including Uganda, Ghana, Malawi, and Rwanda. Organizations like the Wellcome Trust and the UK Department for International Development (DFID) have supported cholera research in Africa, while the US Centers for Disease Control and Prevention (CDC) provides technical expertise [83].

**Figure 5.**
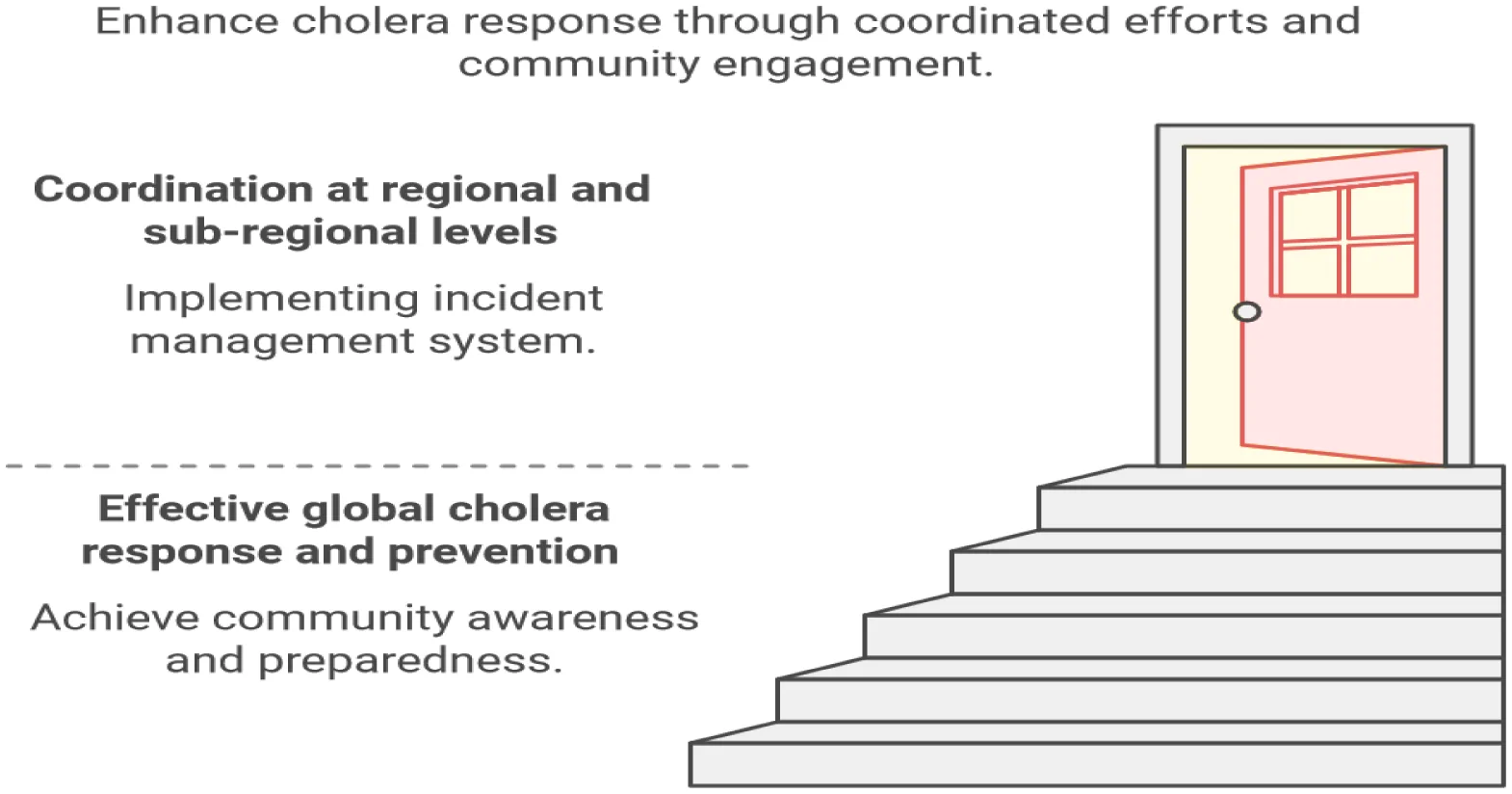
The diagram outlines a stepwise approach to cholera control, beginning with regional planning and Incident Management System (IMS) implementation, and progressing toward community engagement and preparedness. It emphasizes the importance of coordinated, multisectoral strategies to ensure sustainable prevention and response efforts.

At the WHO's regional and subregional offices, coordination takes place at both the regional and subregional levels, with the incident management system (IMS) in action. A preparedness and response plan has been developed within the GTFCC, with participation by relevant stakeholders. The global response plan for addressing cholera includes coordination, early warning and surveillance, case management, provision of essential supplies, logistics, and strategies to prevent the spread of cholera to nearby areas. Advocacy meetings with community leaders and political administrative officials have been organized. Community workers receive training to enhance cholera awareness, and preventive measures are communicated to affected residents through door-to-door outreach and public gatherings [84] (Figure 5).

**Figure 6.**
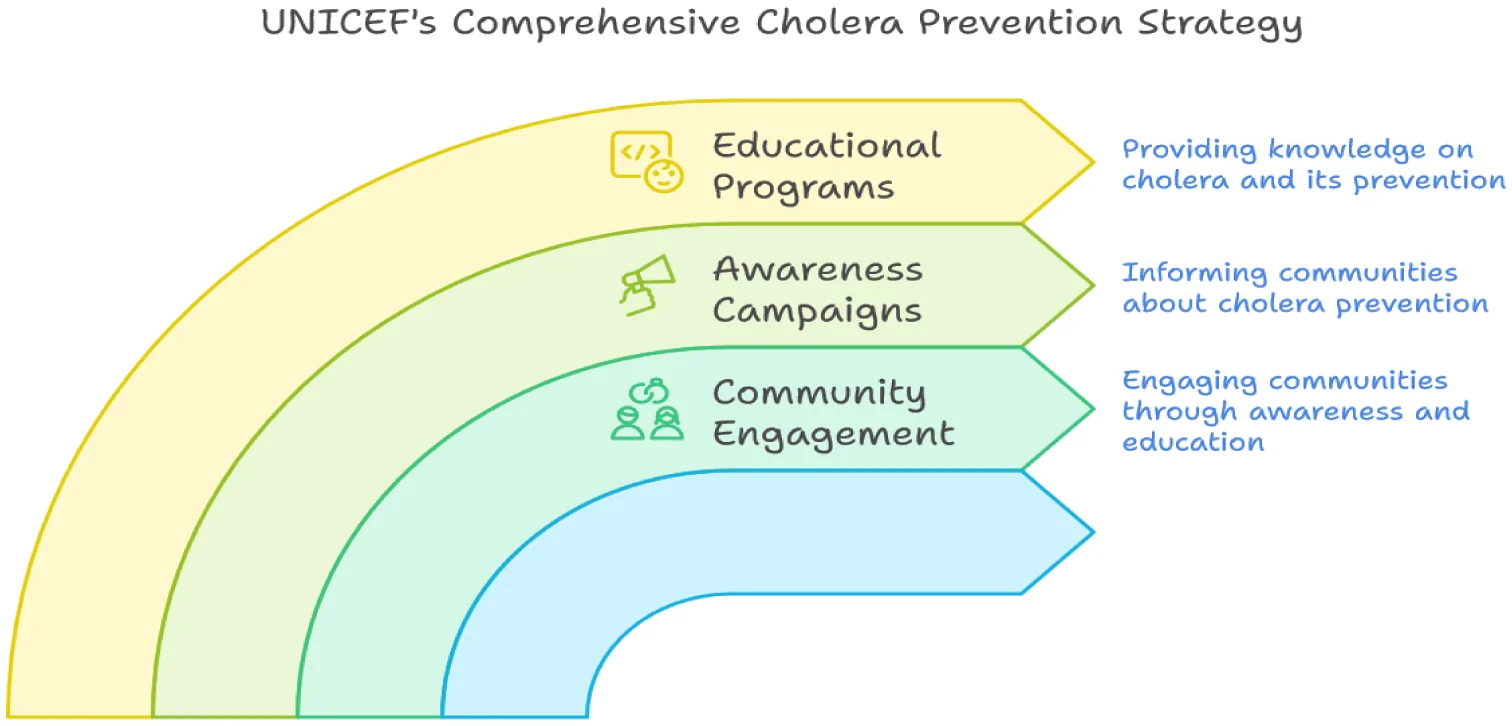
Integrated community-based approach to cholera prevention: UNICEF's strategic framework. This visual illustrates three interconnected pillars educational programs, awareness campaigns, and community engagement that collectively aim to enhance public knowledge, inform communities about cholera prevention, and promote sustained behavioral change through inclusive outreach.

UNICEF significantly contributes to cholera prevention through its WASH programs, which implement sustainable WASH solutions in vulnerable communities and promote access to safe drinking water and basic sanitation. Furthermore, UNICEF emphasizes the importance of community engagement in cholera prevention, supporting awareness campaigns and educational programs to inform communities about the disease and its prevention [85],[86] (Figure 6).

**Figure 7.**
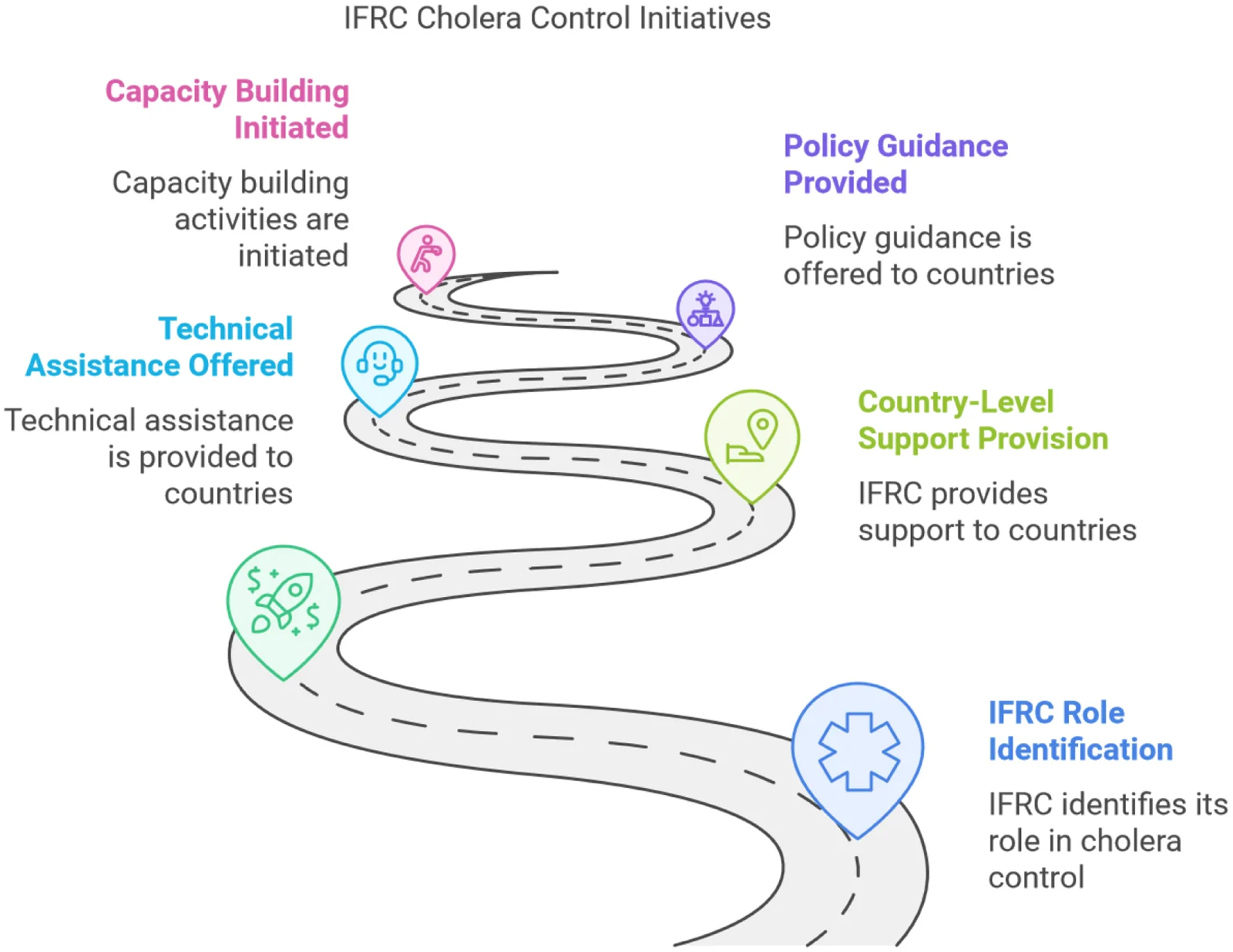
Diagram illustrating the IFRC's phased approach to cholera control. The visual depicts a structured pathway beginning with role identification and progressing through capacity building, technical assistance, country-level support, and policy guidance. This stepwise model underscores the IFRC's coordinated engagement in cholera preparedness and response.

The International Federation of Red Cross and Red Crescent Societies (IFRC) also plays a vital role in cholera prevention and control through its Country Support Platform, which serves as the operational arm of the GTFCC. The IFRC provides country-level support, offering technical assistance, policy guidance, and capacity building. It also implements grassroots-level cholera prevention and response programs, mobilizing volunteers for health education and emergency response efforts [87],[88]. (Figure 7).

Lastly, GAVI, the Vaccine Alliance, contributes to cholera prevention through vaccination efforts, financing the global OCV stockpile, and supporting preventive and emergency vaccination campaigns. GAVI is also focused on raising funds for cholera prevention and control efforts from 2026 to 2030, ensuring that resources are available to combat the disease effectively [89].

## 4. Discussion

The ongoing global challenge posed by cholera underscores several critical lessons in public health practice, policy, and research. These lessons can inform future strategies to combat the disease and guide interventions to mitigate its impact on vulnerable populations.

### 4.1. Importance of robust surveillance systems

One of the key lessons learned is the critical need for robust, real-time surveillance systems to monitor cholera outbreaks and respond promptly. The difficulty in the timely identification and tracking of outbreaks, particularly in regions with limited resources, highlights the gaps in the current surveillance infrastructure. Strengthening data collection, integration, and sharing mechanisms across borders is essential to improving early detection and providing the necessary resources for rapid responses. Moreover, the use of technology, such as AI and machine learning, presents a valuable opportunity to predict and prevent outbreaks by forecasting high-risk periods on the basis of environmental and epidemiological data.

### 4.2. Vaccination gaps and stockpile management

The depletion of the global OCV stockpile, particularly during critical outbreaks, underscores the need for sustainable vaccine production and distribution strategies. The lesson here is twofold. First, vaccination campaigns must be prepared for the inevitable surges in demand during outbreaks; second, vaccine research should focus on developing more durable and cost-effective vaccines, especially for regions with a high cholera burden. The coordination of vaccine distribution through international health organizations like the WHO, UNICEF, and the IFRC should be optimized, ensuring that vaccines reach the most vulnerable populations in the shortest possible time.

### 4.3. Climate change and health risks

The growing recognition of the link between climate change and the spread of cholera serves as a wake-up call for the integration of climate resilience into cholera prevention and control efforts. This lesson highlights the importance of not only addressing the direct effects of cholera transmission, such as water contamination, but also anticipating the indirect impacts of shifting weather patterns on cholera outbreaks. Public health programs should incorporate climate data to develop adaptive strategies that account for changes in the environment, such as floods and droughts, which exacerbate the risk of cholera transmission. Addressing this in the context of broader environmental health policies is key.

### 4.4. Infrastructure and sanitation as long-term solutions

Investment in WASH infrastructure is a long-term strategy that can dramatically reduce the cholera risk. Lessons from past outbreaks show that sustainable access to clean water and waste management solutions is often the most effective prevention method. Efforts should be focused on designing affordable, scalable WASH interventions for resource-limited settings. Furthermore, these interventions must be tailored to the local conditions, considering the specific challenges faced by different communities.

### 4.5. Community engagement and behavior change

Cholera prevention and control programs that fail to adequately involve local communities in both the design and implementation of interventions often experience reduced effectiveness. The importance of community engagement, especially through behavior change communication, has become a central lesson in addressing cholera transmission. For behavior change to be effective, strategies must be context-specific, culturally appropriate, and community-driven. Public health messaging should focus not only on cholera prevention techniques, such as handwashing and sanitation, but also on addressing misconceptions and building trust within the affected communities.

### 4.6. Integrating and strengthening cholera control into broader health systems

Strengthening local health systems, particularly in cholera-endemic regions, is a lesson learned from past experiences, where cholera prevention and control efforts were hampered by weak healthcare infrastructure. To improve cholera prevention and response, efforts must be integrated into broader health systems' strengthening initiatives. This integration helps to build sustainable capacity, enabling countries to handle future outbreaks more effectively. Building a trained workforce, ensuring the availability of necessary health supplies, and establishing emergency response systems are key components of this strategy.

### 4.7. The one health approach

Another key lesson is the necessity of adopting a One Health approach that considers the interaction among human, animal, and environmental health in cholera's transmission. Cholera is often linked to environmental contamination, including the contamination of water sources by both human and animal waste. The One Health approach allows for interdisciplinary research, addressing the zoonotic and environmental factors contributing to cholera. Future research should continue to explore these connections, particularly in regions where human and animal populations overlap.

### 4.8. The economic burden of cholera

A limited understanding of the full economic burden of cholera, including both direct healthcare costs and indirect costs such as lost productivity, remains a significant barrier to effective prevention and treatment. This underscores the need for more comprehensive economic evaluations to inform policy and guide investment in cholera control. Understanding the economic impact of cholera not only justifies the need for increased investments into prevention and treatment but also provides a framework for making more informed decisions about resource allocation in low- and middle-income countries.

### 4.9. International collaboration and coordination

The global nature of cholera outbreaks calls for enhanced international collaboration and coordination. Lessons learned from past cholera epidemics indicate that collective action is crucial, especially when outbreaks cross borders. Strengthening cross-border surveillance, information sharing, and coordinated response plans between countries sharing water resources or geographical boundaries can enhance global preparedness. International cooperation should also extend to humanitarian agencies, NGOs, and local governments to provide timely support during outbreaks.

## 5. Conclusion

The global cholera situation remains a significant public health concern, with the disease continuing to affect numerous countries, particularly in Africa and the Eastern Mediterranean region. Key findings indicate that cholera poses a persistent threat despite advancements in prevention and treatment, leading to substantial morbidity and mortality, especially in resource-limited settings. The burden of cholera is most heavily felt in Africa, which accounts for the majority of global cases and deaths, while the Eastern Mediterranean region has also experienced a significant increase in cases. Multifactorial causes of outbreaks include climate change, ongoing conflicts, inadequate healthcare infrastructure, and poor sanitation. Additionally, the global stockpile of OCVs has been depleted, complicating prevention efforts. International organizations such as the WHO, UNICEF, and the IFRC play crucial roles in coordinating global responses to combat cholera through various initiatives and programs. While there has been a decrease in global case numbers compared with 2023, the alarming increase in deaths highlights the need for continued vigilance and improved interventions.

## 6. Limitations

While this systematic review provides valuable insights into the global cholera situation, several limitations should be considered. The review may be subject to publication bias, as only peer-reviewed, English-language articles were included, potentially excluding relevant non-English studies or unpublished data. Additionally, the heterogeneity of the included studies, varying in their design and outcome measures, may limit the ability to draw generalized conclusions. Inconsistent reporting of economic data and the lack of longitudinal studies also hinder the depth of analysis, particularly regarding the long-term effects of cholera outbreaks. Furthermore, the limited geographic scope, primarily focusing on Africa and the Eastern Mediterranean, may not fully represent the global burden. The exclusion of sociopolitical factors and the variability in data quality also highlight the need for further research in these areas to provide a more comprehensive understanding of cholera's impact and improve prevention strategies.

## 7. Recommendations and future research

To enhance the fight against cholera, several recommendations for future research and policy are necessary. First, enhancing surveillance systems is vital, as developing and implementing robust, real-time surveillance systems will enable timely detection and response to outbreaks. Integrating advanced technologies, such as AI and machine learning, can aid in predictive modeling of cholera outbreaks. Second, investment into vaccine development and distribution is crucial. This includes research for more effective and longer-lasting cholera vaccines, as well as strategies to increase production capacity and improve global distribution mechanisms.

Additionally, prioritizing investments in water and sanitation infrastructure, particularly in high-risk areas, is essential. Researching cost-effective, scalable solutions for clean water access and waste management in resource-limited settings will further support these efforts. Addressing the impact of climate change on cholera transmission is another critical area for study; developing adaptive strategies, and incorporating climate resilience into cholera prevention and control programs will help mitigate the effects of climate change on disease spread.

Community engagement and education should also be emphasized. Developing and evaluating innovative community-based interventions for cholera prevention, as well as researching effective methods for communicating behavior change across diverse cultural contexts, can enhance public health outcomes. Strengthening health systems is another important focus, as integrating cholera prevention and control into broader health system-related strengthening efforts and developing models for sustainable capacity-building in cholera-endemic countries will bolster resilience against future outbreaks.

Adopting a One Health approach is essential to explore the environmental and zoonotic aspects of cholera transmission. Developing interdisciplinary research programs that address the interplay among human, animal, and environmental health in cholera's epidemiology can lead to more comprehensive solutions. Furthermore, analyzing the effectiveness of current global and national cholera prevention and control policies and developing evidence-based recommendations for policy improvements will facilitate rapid and effective responses to outbreaks.

Lastly, conducting comprehensive research on the economic burden of cholera and the cost-effectiveness of various intervention strategies is necessary. Utilizing economic data to advocate for increased investment in cholera prevention and control can mobilize additional resources. Developing frameworks for improved international cooperation in cholera surveillance and response, especially in border regions and among countries sharing water resources, will enhance collective efforts to combat this ongoing public health threat.

## Use of AI tools declaration

The authors declare they have not used AI tools in the creation of this article.


