# Supplementary material for: Cholera outbreaks: Public health implications, economic burden, and preventive strategies
Source: AIMS Public Health. 2025 Aug 5;12(3):767–95. doi: 10.3934/publichealth.2025039 (PMC12538245; doi:10.3934/publichealth.2025039)
Supplement: Supplementary file 1 [file publichealth-12-03-039-s001.pdf]

AIMS Public Health

AIMS Public Health, 12(3): 767–795.
DOI: 10.3934/publichealth.2025039
Received: 23 March 2025
Revised: 26 June 2025
Accepted: 01 July 2025
Published: 05 August 2025

https://www.aimspress.com/journal/aimsph

Review

Cholera outbreaks: Public health implications, economic burden, and preventive strategies

Running title: Cholera outbreaks: Impacts and prevention strategies

Mona Gamal Mohamed*, Eman Abdelaziz Ahmed Dabou, Shaimaa Abdelsamad and Shaimaa Hashem Elsalous

RAK College of Nursing, RAK Medical and Health Sciences University, UAE

* **Correspondence:** Email: mona.mohamed2100@yahoo.com.

Supplementary

Example Search Strategy (PubMed)

The following search string was used for the PubMed database:
("Cholera"[Mesh] OR "cholera outbreaks" OR "cholera epidemic" OR "cholera cases")
AND
("Public Health"[Mesh] OR "health system" OR "health impact" OR "public health response")
AND
("Economic Burden"[Mesh] OR "economic cost" OR "cost-effectiveness")
AND
("Preventive Strategies" OR "cholera prevention" OR "oral cholera vaccine" OR "WASH interventions")
AND
("2019/01/01"[Date - Publication]: "2024/12/31"[Date - Publication])

AIMS Press

© 2025 the Author(s), licensee AIMS Press. This is an open access article distributed under the terms of the Creative Commons Attribution License (https://creativecommons.org/licenses/by/4.0)
